# Virtual reality-based vision therapy versus OBVAT in the treatment of convergence insufficiency, accommodative dysfunction: a pilot randomized controlled trial

**DOI:** 10.1186/s12886-022-02393-z

**Published:** 2022-04-21

**Authors:** Shijin Li, Angcang Tang, Bi Yang, Jianglan Wang, Longqian Liu

**Affiliations:** 1 Department of Optometry, West China Clinical Medical College, Sichuan University, 37 Guoxue Xiang, Chengdu, Sichuan Province China; 2 Department of Ophthalmology, West China Hospital of Sichuan University, 37 Guoxue Xiang, Chengdu, Sichuan Province China

**Keywords:** Vision therapy, Virtual reality, Convergence insufficiency, Accommodative dysfunction, Binocular vision

## Abstract

**Background:**

Virtual reality is being increasingly applied in vision therapy. However, the differences in effectiveness, optimal treatment cycle, and prognosis between virtual reality-based vision therapy and traditional therapies remain unknown. The purpose of this study was to compare the effectiveness of virtual reality-based vision therapy and office-based vergence/accommodative therapy in young adults with convergence insufficiency or accommodative dysfunction.

**Methods:**

The patients were randomly assigned to either the virtual reality-based vision therapy group or the office-based vergence/accommodative therapy group. The vision therapy lasted 12 weeks (1 h/week) in both groups. Binocular visual functions (vergence and accommodation) were measured and a subjective questionnaire-based assessment was performed at baseline and after 6 and 12 weeks of therapy.

**Results:**

In total, 33 patients with convergence insufficiency and 30 with accommodative dysfunction completed the study. After 12 weeks of treatment for convergence insufficiency, the Convergence Insufficiency Symptom Survey score (F_2,31_ = 13.704, P < 0.001), near point of convergence (F_2,31_ = 21.774, *P* < 0.001), positive fusional vergence (F_2,31_ = 71.766, *P* < 0.001), and near horizontal phoria (F_2,31_ = 16.482, *P* < 0.001) improved significantly in both groups. Moreover, the monocular accommodative amplitude (F_2,25_ = 22.154, *P* < 0.001) and monocular accommodative facility (F_2,25_ = 86.164, *P* < 0.001) improved significantly in both groups after 12 weeks of treatment. A statistically significant difference was observed in monocular accommodative facility (F_1,25_ = 8.140, *P* = 0.009) between the two groups, but not in other vergence and accommodative functions (0.098 < *P* < 0.687).

**Conclusion:**

Virtual reality-based vision therapy significantly improved binocular vision functions and symptoms in patients with convergence insufficiency and accommodative dysfunction, thereby suggesting its effectiveness as a new optional or additional treatment for young adults with these conditions.

**Trial registration:**

This study was registered at the Chinese Clinical Trials Registry on 16/04/2019 (identifier: ChiCTR1900022556).

**Supplementary Information:**

The online version contains supplementary material available at 10.1186/s12886-022-02393-z.

## Introduction

Binocular vision is important for hand–eye coordination [1], reading [2], stereopsis [3], and academic performance [4]. Convergence insufficiency or accommodative dysfunction can affect normal binocular vision functions. When patients with convergence insufficiency view a near visual target, the two eyes do not converge all the way, in the case of exophoria. They exhibit several symptoms, such as sore eyes, eye pain, headache, blurred vision, and double vision [5, 6]. Patients with accommodative dysfunction have difficulty focusing on targets and may experience blurred vision, headache, and fatigue associated with tasks at a near viewing distance and holding reading material close or away [7, 8]. The prevalence of convergence insufficiency and accommodative dysfunction is approximately 2.7–17.6% [6, 9–12] and 2.3–20.2% [8, 13], respectively, in the general population. Both conditions have been shown to cause asthenopia, whose prevalence has increased owing to the widespread use of electronics and prolonged near work, especially among young adults [14, 15].

Previous studies on children and adults have shown that vision therapy can effectively improve binocular vision functions and relieve asthenopia. The Convergence Insufficiency Treatment Trial (CITT) study group [16–20] reported that office-based vergence/accommodative therapy (OBVAT) was more effective than home-based push-up therapy, computer-based vision therapy, and office-based placebo therapy for improving binocular function in patients with convergence insufficiency. Other studies [21, 22] also showed that OBVAT produced significant improvements in monocular accommodative amplitude and facility. Some studies [23, 24] that investigated the effects of OBVAT on attention and reading in children with convergence insufficiency found that it could improve reading comprehension, reading speed, and academic behavior. The improvements in attention and reading were highly consistent with improvements in binocular functions. Therefore, vision therapy is important for patients with convergence insufficiency.

However, the implementation of OBVAT requires the guidance of a professional optometrist. Additionally, many patients do not have the conditions to receive OBVAT, such as being too busy to work, too far from the hospital, and so on. Previous studies have shown the effect of home-based therapy is not good as that of office-based therapy [25], which may be due to the lack of professional guidance, and boredom leads to low compliance.

Virtual reality (VR) has been implemented in vision therapy such as amblyopia. Studies have shown that virtual reality training improves not only amblyopia treatment compliance [26] but also the visual acuity of the amblyopic eye in adults with anisometropic amblyopia [27]. A few studies have also used virtual reality for treating convergence insufficiency and accommodative dysfunction. Yaramothu et al. [28] found that virtual reality therapy effectively improved the vergence function and relieved symptoms of convergence insufficiency. Moreover, Boon et al. [29] found that a virtual reality therapy group had higher compliance when patients with convergence insufficiency were trained using anaglyphs and a virtual reality game of snakes. Munsamy et al. [30] reported that virtual reality gaming for 25 min improved accommodative and vergence facility. These studies suggested the utility of virtual reality-based vision therapy for treating convergence insufficiency and accommodative dysfunction.

With the development of technology, VR will be more and more widely used. This study aimed to compare the effectiveness of virtual reality-based vision therapy and OBVAT for young adults with convergence insufficiency and accommodative dysfunction. Since VR does not require experienced optometrists to be fully involved in the training process, VR may be a new option for visual training, especially in applications such as the home. Through programmed training settings and remote guidance patients’ compliance and effectiveness can be improved.

## Methods

### Study design

**Fig. 1:**
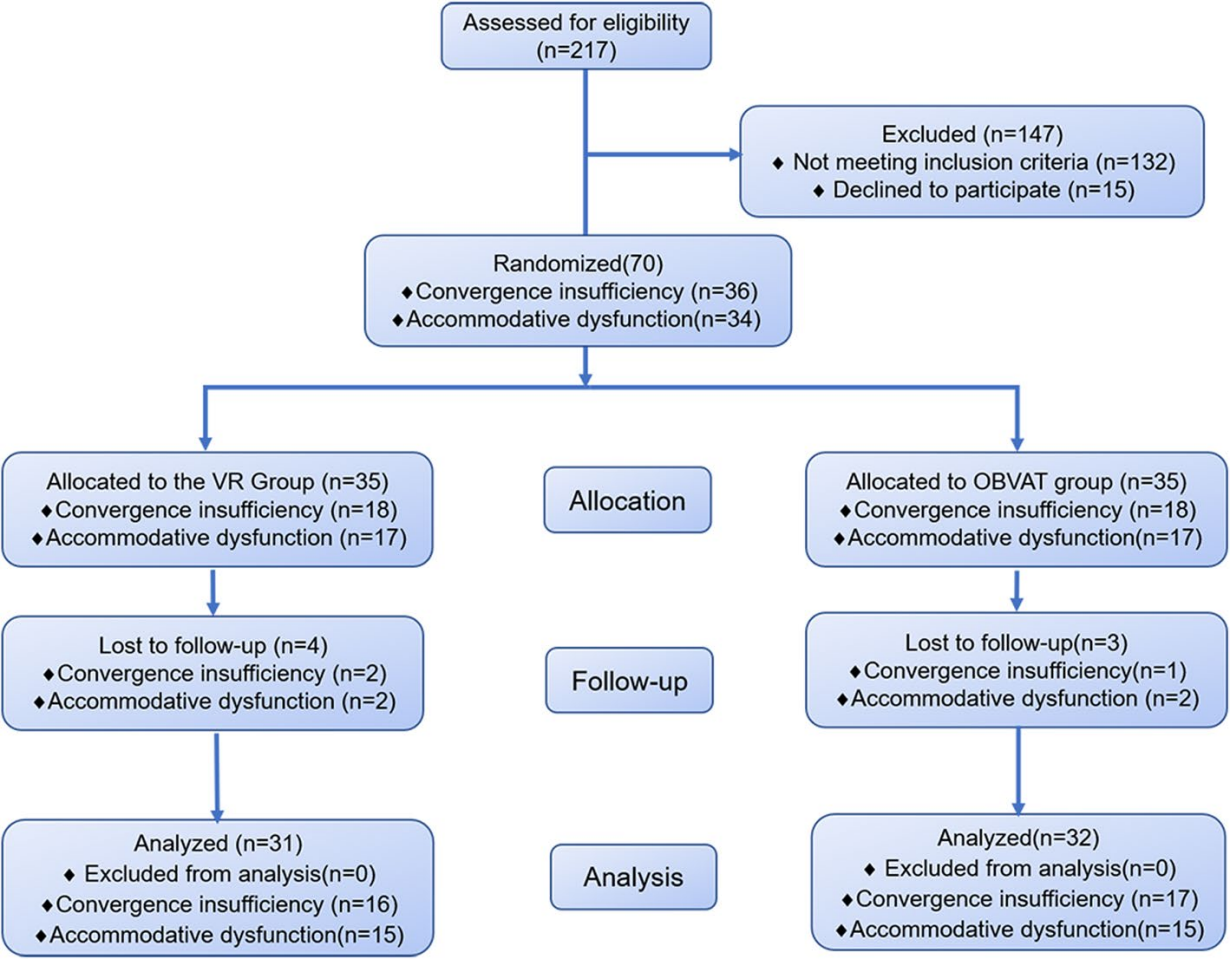
Flowchart showing study completion for each group. VR: virtual reality; OBVAT: office-based vergence/accommodative therapy

In this single-center, prospective, longitudinal trial, patients with convergence insufficiency or accommodative dysfunction were randomly allocated to either the virtual reality or OBVAT group in a 1:1 ratio using a random allocation sequence generated with the Clinical Trial Management Public Platform. The pre- and after-training outcomes were assessed by a masked examiner. Figure 1 presents the study flowchart.

### Patients

The patients were enrolled between May 2019 and October 2020 at the Department of Ophthalmology, West China Hospital of Sichuan University. The study was approved by the West China Hospital of Sichuan University Biomedical Research Ethics Committee. The study conformed to the tenets of the Declaration of Helsinki. The patients signed informed consent before any study procedures were performed. The inclusion, exclusion, and diagnostic criteria are listed in Table 1.

**Table 1:** Inclusion criteria, exclusion criteria, and diagnostic criteria

**Inclusion criteria**
Age 18–35 years
Best correct visual acuity of 20/25 or better in each eye at distance (5 m) and near (40 cm)
Random-dot stereopsis better than or equal to 480 s of arc (40 cm)
No previous prism or near add before study enrollment
Willing to wear appropriate refractive correction for at least 2 weeks before vision therapy (spectacles are required for diopters that meet the following criteria) Myopia ≤ -0.75 D spherical equivalent in either eye Hyperopia ≥ + 2.00 D spherical equivalent in either eye Anisometropia ≥ 0.75 D spherical equivalent Astigmatism ≥ 1.00 D in either eye
**Exclusion criteria**
≥ 2^△^ esophoria at distance
History of strabismus, amblyopia, manifest or latent nystagmus History of vision therapy
Organic lesions of the eye
History of any ocular surgery
History of head trauma or known disease of the brain
Systemic or mental diseases, such as diabetes or anxiety, affecting accommodation, vergence, and ocular motility
Use of ocular or systemic medications containing atropine, pirenzepine, or antiepileptic in the past 3 months
**Diagnostic criteria**
**Convergence Insufficiency** We enrolled participants with convergence insufficiency who had a near exophoria at least 4△ greater than distance exophoria and met at least two of the following three criteria:
1) Near point of convergence break point ≥ 6 cm
**2**) Reduced near positive fusional vergence(break point ≤ 15△ or failed Sheard’s criterion)
**3**) Convergence Insufficiency Survey Score ≥ 21 points
**Accommodative Dysfunctions** We enrolled participants with accommodative dysfunction who met at least one of the following two criteria:
1) The monocular amplitude of accommodation ≥ 2 diopters below the minimum prediction(15-age/4)
2) Monocular accommodative infacility ≤ 6 cycles per minute with ± 2.00 D lenses

The sample size of convergence insufficiency was calculated based on the outcome measures reported in the previous study of VR and the CITT Manual of Procedures. The mean improvements in the CISS score after 12 weeks were 16.2 and 10, respectively. In addition, the predicted standard deviation was 5.5. Power analysis (power of 80% and significance level of 5%) indicated that 14 participants were needed for each group, and we estimated that approximately 20% of participants might withdraw. Therefore, a total of 18 participants with convergence insufficiency were randomly assigned to each treatment group.

### Ocular health examination

Slit-lamp microscopy, scanning laser ophthalmoscopy, and tonometry examinations were used to exclude patients with eye disease. Sodium fluorescein paper was used to test the tear break-up time and exclude patients with dry eye.

### Clinical outcome measures

The patients received binocular vision examinations and completed subjective questionnaires (Convergence Insufficiency Symptom Survey, CISS) at baseline and after 6 and 12 weeks of therapy. The examination could not be performed during the same day as the training, but it was completed within 1 week after the last training. Binocular vision functions were tested under best-corrected refraction. The primary outcome measure of convergence insufficiency was the CISS score, and the secondary outcome measures were near point of convergence (NPC), positive fusional vergence (PFV), and near horizontal phoria. The primary outcome measures of accommodative dysfunction were monocular accommodative amplitude and facility.

The parameters examined in this study are as follows.

1. Phoria and AC/A ratio
  The distance and near horizontal heterophoria were determined by both the cover test and the von Graefe technique. The target used for both methods was an isolated letter 20/30 at distance (5 m) and near (40 cm). The accommodative convergence to accommodation (AC/A) ratio was measured through the gradient and the calculated methods. The gradient testing will be performed at near (40 cm). The phoria was measured after adding +1.00 (-1.00) lenses OU in addition to distance prescription.
2. Measurement of vergence function
  NPC was measured using the Astron Accommodative Rule (Bernell, Mishawaka, IN) with an isolated 20/30 letter at 40cm. The target moved toward the participant’s nose, and the distance was recorded when diplopia is reported (or a deviation of the participant’s eyes was observed). Positive and negative fusional vergence were measured by rotary prisms with a single column of 20/30 letters at distance (5 m) and near (40 cm). The blur, break, and recovery points were measured.
3. Measurement of accommodative function
  Negative and positive relative accommodation (NRA and PRA) were measured with a single column of 20/30 letters at 40 cm. NRA was tested before PRA to avoid straining participants’ accommodation by negative lenses, leading to overestimation of NRA. The monocular and binocular accommodative amplitudes were measured with the Accommodative Rule (with a column of 20/30 letters at 40 cm). The monocular and binocular accommodative facilities were measured with ±2.00 D flipper lenses and a 20/30 near chart at 40 cm. Binocular cross-cylinder was used to assess the binocular accommodative response.
4. Convergence Insufficiency Symptom Survey

### Treatment

All therapies were conducted under best-corrected refraction.

### Virtual reality-based vision therapy group

**Fig. 2:**
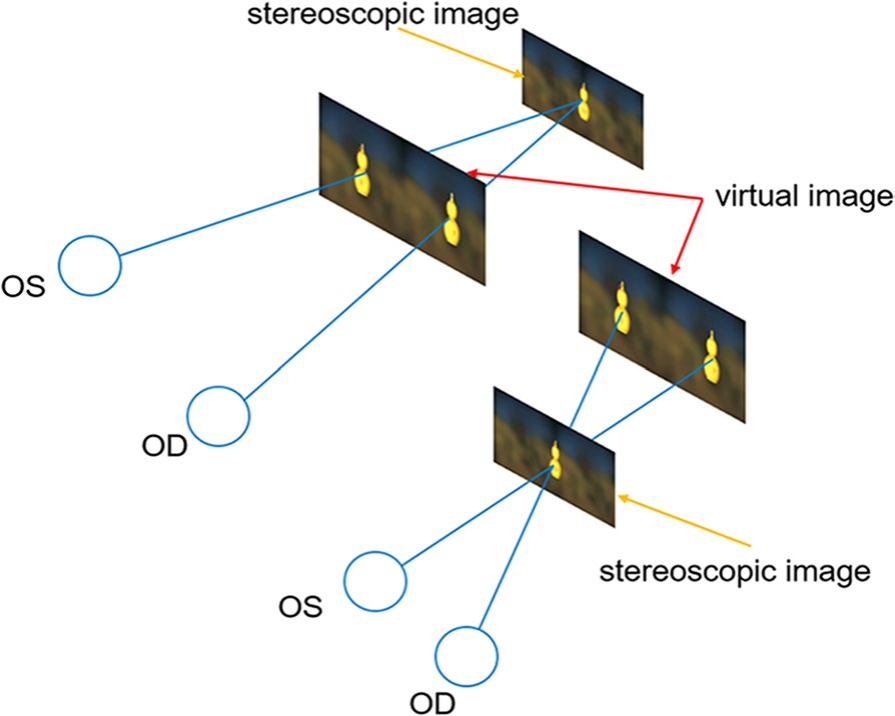
A simplified schematic representation of the VR imaging principles

Technology Co., Ltd). Virtual reality-based vision therapy was based on the principle of dividing two similar images (targets) into both eyes. This was accomplished by dissociating the eyes with two screens (Fig. 2). The size of the head-mounted display was 184.6 × 78.1 × 91.8 mm with adjustable pupil distance (56-72 mm). The screen resolution was 1920 × 1080 pixels, and the display refresh frequency was 120 Hz.

#### Virtual reality-based vision therapy for vergence

##### Stimuli

**Fig. 3:**
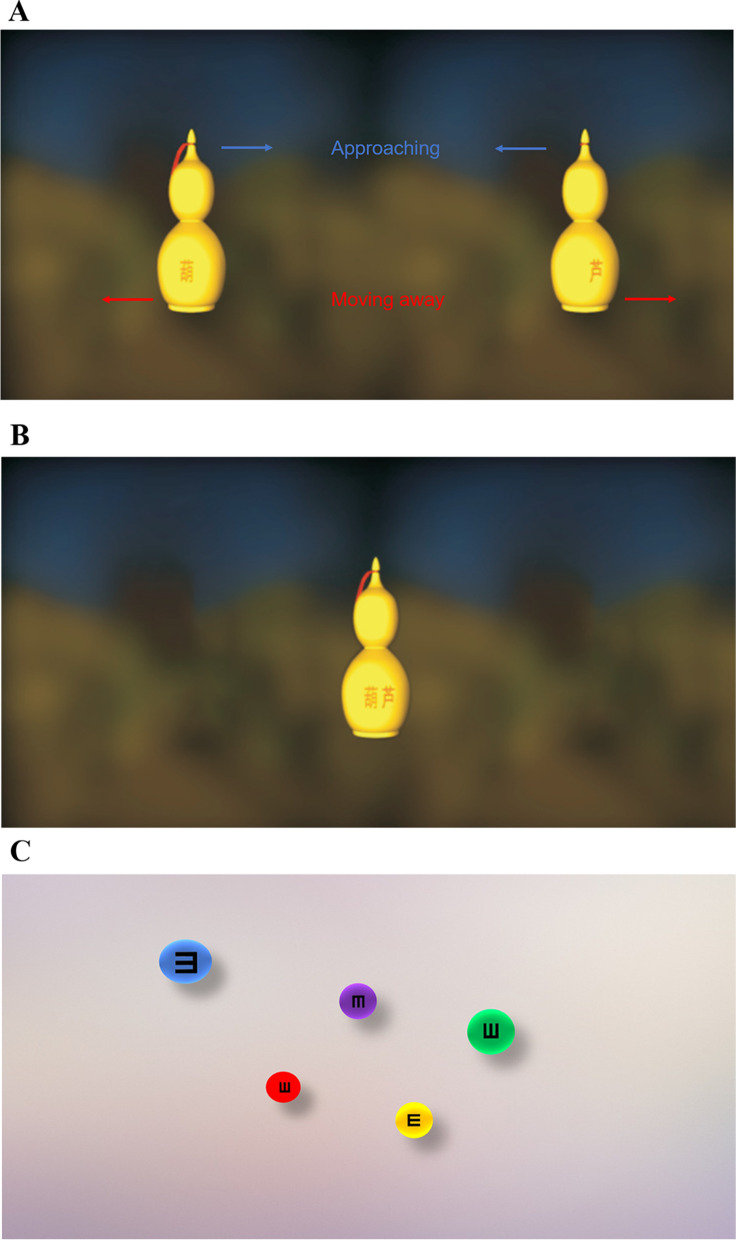
The schematic diagram of VR therapy

Visual targets presented on the virtual reality screen were horizontally separated and induced vergence demands. The targets were two similar images for two eyes and included human figures, animal figures, or objects (e.g., gourds) (Fig. 3A). The background was blurred to highlight the target at the centre. The two images approached or moved away simultaneously to change the vergence demands, which ranged from 20^△^ base-in to 30^△^ base-out, and the eyes were trained for both convergence and divergence.

##### Task

The patients were asked whether they saw a figure that appeared to be floating closer than the plane of the screen (Fig. 3B). The targets, which were selected by the therapist, varied in their level of complexity, thereby affecting the ability of the observer to fuse the images properly. For instance, visual targets with simple lines, larger areas, and less fine detail were easier to fuse, whereas those with complex shapes and finer detail were more difficult to fuse.

The patients first needed to fuse the two targets into one at the zero prismatic demand position. The instrument then increased the prismatic demand at 2 PD per second. To ensure that the patients received proper training during the therapy, they had to maintain a clear, fused vision. The suppression cues of the targets were two characters (Fig. 3A and B) which were separately presented to each eye. If they saw a double image, only one character, and/or lost depth perception, they immediately reported it to the therapist, who would restart the vergence demand from zero.

Figure 3A shows the images seen by each eye, with the two images moving simultaneously to produce a converging or diverging stimulus. Figure 3B shows the complete stereo image after fusion. Figure 3C shows balls of different sizes and colors with optotypes appearing on the screen. In training sequences, balls appeared on the screen one by one.

##### Training procedure

There were 15 trials with different targets and different vergence demands. At each visit, the patients performed 4 to 5 procedures. Each training session lasted approximately 8 to 10 min, with a short break between sessions.

#### Virtual reality-based vision therapy for accommodation

##### Stimuli

In the training sequences, balls of different sizes and colours appeared sequentially on the screen (Fig. 3C). The optotype on the balls was the letter ‘E’ at different sizes, as is the case in the visual acuity chart. The background was blurred to highlight the targets. The balls were moved from far to near or vice versa by changing the binocular parallax. A ball with an optotype appeared on the screen every 1.5 to 3 s.

##### Task

During ball movement, the patients were required to always keep the optotype clear. Usually, the task began with slower moving balls and larger targets. To ensure that the patients received proper training during the therapy, they had to maintain clear vision. If the patients saw a blurred optotype, they immediately reported it to the therapist and proceeded with the training protocol after a short break.

##### Training procedure

There were 8 trials with different sequences of targets and ball movement speeds. Each session lasted approximately 5 to 8 min, with a short break between sessions. The VR based therapy was given in-office. The duration of the therapy was 12 weeks(1 h/week).

All patients in the VR group did both accommodative and vergence therapy according to each patient’s situation and training progress.

### OBVAT group

The treatment followed the training strategy of improving fusional vergence (smooth and jump vergence) for CI patients [31]. The OBVAT procedures were divided into three phases. Phases 1 and 2 included the Brock string and Barrel card tests for cross convergence and vectograms, LifeSaver cards, aperture rule, and eccentric circles for fusional vergence. The fusional vergence ranges were set as the same as that in VR group. Phase 3 mainly focused on jump vergence and added prism facility. For accommodative therapy, phases 1 and 2 included monocular letter chart, monocular lens sorting, and monocular accommodative facility. Phase 3 added binocular accommodative facility. Personalized training programs were used according to each patient’s situation and training progress and all patients in the OBVAT group did both accommodative and vergence therapy.

### Statistical analysis

All data analyses were performed using IBM SPSS Statistics for Windows, Version 23.0 (IBM Corp., Armonk, NY). A chi-square test was used to compare sex differences between the groups. An independent t-test was used to compare the baseline findings between the groups. Repeated-measures analysis of variance was used to determine any significant effect of visit and group on binocular functions and symptoms (within-subjects factor: duration of treatment, between-subjects factor: treatment group). The Pearson chi-square test was used to compare the proportion of each indicator reaching normal in the two groups. *P* values < 0.05 were considered statistically significant.

## Results

This study enrolled 36 patients (VR: 23.63 ± 1.75 years, OBVAT: 23.53 ± 2.00 years, mean ± SD) with convergence insufficiency and 34 (VR: 23.07 ± 1.87 years, OBVAT: 23.13 ± 2.03 years, mean ± SD) with accommodative dysfunction; 7 patients (3 with convergence insufficiency and 4 with accommodative dysfunction) were lost to follow-up. Finally, 33 (91.67%) patients with convergence insufficiency and 30 (88.24%) with accommodative dysfunction completed the study. The demographics and baseline data of the patients are shown in Table 2.

**Table 2:** Study population demographics and clinical measures at baseline

	**Virtual reality-based vision therapy,** **mean ± SD**	**Office-based vergence/accommodative therapy, mean ± SD**	***P* value**
**Convergence insufficiency**			
Sex, M: F	5:11	6:11	1.000
Age (year)	23.63 ± 1.75	23.53 ± 2.00	0.885
Right eye spherical equivalent, (D)	-3.91 ± 1.97	-3.60 ± 2.29	0.680
Left eye spherical equivalent, (D)	-3.67 ± 1.94	-2.88 ± 2.41	0.310
Phoria at distance (cover test) (△)	-3.75 ± 3.62	-2.88 ± 2.76	0.443
Phoria at near (cover test) (△)	-16.31 ± 3.99	-15.35 ± 2.83	0.430
NPC break (cm)	6.60 ± 3.63	6.00 ± 2.95	0.763
Gradient AC/A ratio	2.44 ± 2.21	2.68 ± 2.29	0.979
Calculated AC/A ratio	1.59 ± 1.40	1.61 ± 1.16	0.609
PFV break at near, (△)	22.53 ± 6.15	23.06 ± 5.18	0.828
PFV recovery at near, (△)	9.03 ± 8.85	9.88 ± 7.31	0.765
CISS score	27.69 ± 8.46	25.18 ± 9.40	0.427
**Accommodative dysfunction**			
Sex, M: F	7:8	5:10	0.710
Age (year)	23.07 ± 1.87	23.13 ± 2.03	0.926
Right eye spherical equivalent, (D)	-4.37 ± 1.90	-3.90 ± 2.06	0.525
Left eye spherical equivalent, (D)	-4.03 ± 1.83	-3.43 ± 2.40	0.447
NRA, (D)	2.38 ± 0.40	2.47 ± 0.25	0.583
PRA, (D)	-2.90 ± 1.75	-2.59 ± 0.65	0.644
BCC, (D)	0.06 ± 1.06	0.34 ± 1.02	0.596
Monocular accommodative amplitude, (D)	10.94 ± 3.76	10.41 ± 2.66	0.750
Monocular accommodative facility, (cpm)	6.31 ± 2.56	4.94 ± 4.44	0.461

*NPC* near point of convergence, *AC/A* accommodative convergence to accommodation, *PFV* positive fusional vergence, *CISS* Convergence Insufficiency Symptoms Survey, *NRA* negative relative accommodation, *PRA* positive relative accommodative, *BCC* binocular cross-cyclinder, *D* diopters, △ prism diopters, *cpm* cycles per minute

No significant intergroup differences were observed in age, refraction, horizontal phoria at distance or near, NPC, gradient or calculated ratio of accommodative convergence to accommodation, PFV, CISS scores, negative relative accommodation, positive relative accommodation, or binocular cross-cylinder.

### Changes in symptoms and vergence function

The clinical measures of patients with convergence insufficiency at baseline and after 6 and 12 weeks are presented in Table 3.

**Table 3:** Changes in outcome measures of patients with convergence insufficiency by treatment group

CISS score	Baseline	6 weeks	12 weeks	Total change
VR group	27.69 ± 8.50	22.63 ± 9.88	21.75 ± 9.13	-5.94 ± 3.11
OBVAT group	25.18 ± 9.40	18.82 ± 8.15	16.47 ± 8.02	-8.71 ± 2.99
Effect size	group	group*time	time	
*F* value	2.298	0.424	13.074	
*P* value	0.140	0.656	< 0.001	
NPC break (cm)	Baseline	6 weeks	12 weeks	Total change
VR group	6.59 ± 3.63	5.06 ± 2.73	4.62 ± 2.38	-1.97 ± 1.09
OBVAT group	6.00 ± 2.95	3.91 ± 1.99	3.82 ± 1.41	-2.18 ± 0.80
Effect size	group	group*time	time	
*F* value	1.084	0.478	13.290	
*P* value	0.306	0.624	< 0.001	
PFV blur or break at near (△)	Baseline	6 weeks	12 weeks	Total change
VR group	22.63 ± 6.15	32.97 ± 7.98	34.47 ± 5.20	11.84 ± 2.01
OBVAT group	23.06 ± 5.18	34.68 ± 5.98	35.47 ± 4.64	12.41 ± 1.69
Effect size	group	group*time	time	
*F* value	0.421	0.008	61.222	
*P* value	0.521	0.888	< 0.001	
Near horizontal phoria (△)	Baseline	6 weeks	12 weeks	Total change
VR group	-16.31 ± 3.99	-13.94 ± 4.60	-13.13 ± 5.15	3.19 ± 1.63
OBVAT group	-15.35 ± 2.83	-11.53 ± 5.62	-9.94 ± 6.33	5.41 ± 1.68
Effect size	group	group*time	time	
*F* value	2.261	1.067	16.482	
*P* value	0.143	0.350	< 0.001	

*CISS* Convergence Insufficiency Symptoms Survey, *NPC* near point of convergence, *PFV* positive fusional vergence, △ prism diopters

The repeated-measures analysis of variance showed a significant effect of visit on the CISS score (F_2,31_ = 13.704, *P* < 0.001), NPC break (F_2,31_ = 21.774, *P* < 0.001), PFV break (F_2,31_ = 71.766, *P* < 0.001), and near horizontal phoria (F_2,31_ = 16.482, *P* < 0.001). To control for the baseline differences between the groups, the measures at 6 and 12 weeks were normalized to the baseline values (ex. 12-week data – baseline data), but the repeated-measures analysis of variance also showed no statistically significant effect of group on the measures of the CISS score (F_1,31_ = 0.437, *P* = 0.514), NPC break (F_1,31_ = 0.258, *P* = 0.615), PFV break (F_1,31_ = 0.166, *P* = 0.687), and near horizontal phoria (F_1,31_ = 1.484, *P* = 0.232). Moreover, the post hoc comparison showed no statistically significant effect of 6 and 12 weeks of therapy in either group (0.237 < *P* < 0.675).

After 12 weeks of therapy, 81.3% of patients in the virtual reality group and 88.2% in the OBVAT group had a NPC less than 6. In the VR group, 93.75% (15/16) patients failed Sheard’s criteria before therapy, and 80% (12/15) of them achieved the criteria after the therapy. In the OBVAT group, 94.12% (16/17) patients failed Sheard’s criteria before therapy, and 87.5% (14/16) of them achieved the criteria after the therapy. The difference in the proportion of NPC and PFV reaching normal between the two groups was not statistically significant. Only 9 patients in the virtual reality group and 11 in the OBVAT group had Convergence Insufficiency Symptom Survey scores smaller than 21.

### Changes in accommodative function

In this study, only the amplitude and facility of the right eye were reported because the left eye and binocular amplitude and facility had a similar tendency to those of the right eye.

The clinical measures of patients with accommodative dysfunction at baseline and after 6 and 12 weeks are presented in Table 4. The repeated-measures analysis of variance showed a significant effect of visits on monocular accommodative amplitude (F_2,25_ = 22.154, *P* < 0.001) and facility (F_2,25_ = 86.164, *P* < 0.001). To control for baseline differences between the groups, measures at 6 and 12 weeks were normalized to baseline values. The repeated-measures analysis of variance also showed no statistically significant effect of group on the measures of monocular accommodative amplitude (F_1,25_ = 2.958, *P* = 0.098), but showed a statistically significant difference in monocular accommodative facility between the two groups (F_1,25_ = 8.140, *P* = 0.009). Moreover, the post hoc comparison showed a statistically significant difference in monocular accommodative amplitude (*P* = 0.002) and facility (*P* < 0.001) between 6 and 12 weeks of therapy.

**Table 4:** Changes in outcome measures of patients with accommodative dysfunctions by treatment group

Monocular accommodative amplitude, (D)	Baseline	6 weeks	12 weeks	Total change
VR group	10.94 ± 3.76	12.40 ± 3.41	13.86 ± 3.35	2.91 ± 1.78
OBVAT group	10.41 ± 2.66	12.64 ± 3.28	15.15 ± 2.79	4.73 ± 1.36
Effect size	Group	group*time	time	
*F* value	0.035	1.324	27.202	
*P* value	0.853	0.274	< 0.001	
Monocular accommodative facility, (cpm)	Baseline	6 weeks	12 weeks	Total change
VR group	6.31 ± 2.56	13.38 ± 5.62	16.81 ± 7.34	10.50 ± 2.75
OBVAT group	4.94 ± 4.44	12.75 ± 4.74	17.25 ± 2.58	12.31 ± 1.82
Effect size	Group	group*time	time	
*F* value	0.631	1.636	86.164	
*P* value	0.434	0.213	< 0.001	

*D* diopters, *cpm* cycles per minute

After 12 weeks of therapy, one patient in the virtual reality group and one in the OBVAT group still had monocular accommodative amplitudes less than the minimum prediction (15—age/4) after 12 weeks of therapy. While 5 patients in the virtual reality group still had monocular accommodative facility less than 12 cpm, none in the OBVAT group did. The Pearson chi-square with Fisher’s exact test showed a statistically significant intergroup difference in effectiveness in reaching the normal monocular accommodative facility (*P* = 0.042).

## Discussion

In this study, the OBVAT group was chosen as the control group because OBVAT is the most effective therapy currently known for improving both binocular vision and accommodative functions in those patients [16, 21]. Previous studies [28, 29] have shown that virtual reality-based vision therapy is promising, and the results of the present study suggest that virtual reality training can be an effective method for patients with convergence insufficiency and accommodative dysfunction.

### Changes in symptoms and vergence function in patients with convergence insufficiency

After 12 weeks of treatment, the CISS score, NPC, PFV, and near horizontal phoria improved significantly in both the virtual reality and OBVAT groups. The proportion of achieving normal NPC and PFV in the virtual reality and OBVAT groups was 81.3% and 88.2%, respectively. The difference between the two groups was not significant. Therefore, virtual reality-based vision therapy can be as effective as OBVAT in patients with convergence insufficiency.

In this study, the mean improvements in positive fusional vergence were 11.8 and 13.5^△^ base-out in the virtual reality and OBVAT groups, respectively, and these were similar to the values reported in two other studies [28, 29] that used virtual reality therapy for adults with convergence insufficiency (3.9 and 14.7^△^ base-out, respectively). However, the mean improvement was less than that reported in previous adult studies [17, 32, 33] using OBVAT for vision therapy. One reason for this could be the difference in refractive errors. The patients in this study had a higher degree of myopia (mean refractive error of the right eye: -4.02 D). In the studies by Singh et al. [33] and Alvarez et al. [32], the proportion of patients with myopia was only approximately 25%. In an adult study of CITT [17], the mean refractive error of the right eye was -0.92 D. Patients with a higher degree of myopia might exhibit a higher level of near PFV and closer NPC than those with a lower degree of myopia [34]. Therefore, the baseline values in the present study were higher than those in previous studies, and these better baseline values potentially resulted in relatively less improvement. The second reason could be that neither group received home reinforcement therapy. Previous studies [16, 25] have found better results in groups receiving home reinforcement therapy.

Similarly, the mean improvement in the NPC was 2.08 and 2.18 cm in the virtual reality and OBVAT groups, respectively, which was similar to the 2.4 cm improvement reported in a previous study [29] that also used virtual reality therapy for adults with convergence insufficiency. However, the mean improvement in near point of convergence was less than that in previous adult studies [17, 32, 33] using OBVAT. This could also be attributed to the better baseline values and the absence of home reinforcement therapy in the present study groups.

However, the mean improvements in the CISS score in the present study were 5.93 and 8.71 points for the virtual reality and OBVAT groups, respectively. These values were less than those reported in a previous adult study of CITT [17]. Moreover, only 9 patients in the virtual reality group and 11 in the OBVAT group had CISS scores smaller than 21. The main reason for this could be that even during vision therapy, some patients still spent much time in near work using a computer or mobile phone. This suggests the need for changes in life and work habits to achieve improvements in symptoms. Moreover, the CISS was developed 20 years ago, and its validity may have decreased because the widespread use of electronic devices and long durations of near work have become important causative factors for asthenopia [35–37]. This also suggested that in future studies, life and work habits should be quantified and included as measurement factors to evaluate the effectiveness of vision therapy.

To summarize, virtual reality-based vision therapy can offer benefits to patients with convergence insufficiency. Targeted stimuli in virtual reality allowed for the presentation of hierarchical and repetitive stimuli. Meanwhile, stimuli, such as vergence demands and binocular disparity, were modulated during vision therapy.

### Changes in accommodation in patients with accommodative dysfunction

After 12 weeks of treatment, monocular accommodative amplitude and facility improved significantly in the virtual reality and OBVAT groups. The mean improvement and proportion of achieving normal monocular accommodative facility were statistically significant in the two groups, but those for monocular accommodative amplitude were not significant. Therefore, virtual reality-based vision therapy can be effective for patients with accommodative dysfunction. Nevertheless, its effect on improving accommodative facility was lower than that of OBVAT.

The mean improvements in monocular accommodative facility (9.8 and 12.5 cpm in the virtual reality and OBVAT groups, respectively) observed in the present study were consistent with the improvements (9.4 and 10.9 cpm) reported in previous studies [21, 38] on children.

In this study, we found that VR therapy could improve subjects’ accommodative amplitude and accommodative facility compared to baseline, and this was similar to the previous studies. In Munsamy’s study [39], they analyzed that the improvements may be due to the vergence accommodation and accommodative-vergence capability of adaptation in a VR environment. In the VR environment, the accommodative plane keeps static, while the vergence plane keeps changing. This contributes to the conflict between accommodation and vergence system [40]. In addition, the change of brightness during training caused the change of microfluctuations and pupil size, which also changed the accommodation [41].

However, the improvement of accommodative function in the VR group was less than that in the OBVAT group. This may be due to different training strategies used in VR and OBVAT groups for accommodation. In OBVAT group, lenses with different diopters and reading materials at different distances were used to stimulate or relax accommodation. Thus, it is worthy to try to incorporate a variable lens into the VR headset and investigate the effectiveness of training in future study.

The mean improvements in monocular accommodative amplitude in the virtual reality and OBVAT groups were less than those reported in previous studies on children [21]. In addition to the larger baseline values in this study, age was an important factor affecting the improvement in accommodative amplitude. Moreover, improvements in accommodative function may require a longer duration than improvements in convergence insufficiency. Therefore, improving the effectiveness of virtual reality-based vision therapy for accommodative dysfunction needs further investigation.

### Limitations

This study has several limitations. First, this study did not assess other factors that might affect fatigue symptoms, such as the occupational background and near work habits of the included patients. Second, no placebo control group or a group that was followed for natural history was set in this study, and repeated measurements could also have induced training effects. Finally, a larger sample size and longer follow-up time may be needed in future research to confirm these findings.

## Conclusion

Virtual reality-based vision therapy significantly improved binocular functions and symptoms in patients with convergence insufficiency and accommodative dysfunction. It may be a new optional or additional treatment for young adults with convergence insufficiency and accommodative dysfunction. Moreover, virtual reality-based vision therapy still needs to be optimized, diversified and personalized to improve the training effectiveness and shorten the response time.

## Supplementary Information

**Additional file 1.**

## Data Availability

Data sharing is not applicable to this article as no datasets were generated or analyzed during the current study.

## Abbreviations

CITT: Convergence Insufficiency Treatment Trial
OBVAT: Office-based vergence/accommodative therapy
VR: Virtual reality
CISS: Convergence Insufficiency Symptom Survey
NPC: Near point of convergence
PFV: Positive fusional vergence
