# Supplementary material for: Virtual reality-based vision therapy versus OBVAT in the treatment of convergence insufficiency, accommodative dysfunction: a pilot randomized controlled trial
Source: BMC Ophthalmol. 2022 Apr 21;22:182. doi: 10.1186/s12886-022-02393-z (PMC9027290; doi:10.1186/s12886-022-02393-z)
Supplement: Supplementary file 1 — Additional file 1. [file 12886_2022_2393_MOESM1_ESM.docx]

**Table 1. Outcome measures of patients with convergence insufficiency at 12 weeks by treatment group**

| **Outcome measure** | **VR group (95% CI)** | **OBVAT group (95% CI)** |
| --- | --- | --- |
| **CISS score** |  |  |
| Baseline | 27.69 (23.18, 32.20) | 25.18 (20.35, 30.01) |
| 12 weeks | 21.75 (16.88, 26.62) | 16.47 (12.35, 20.60) |
| Total change | -5.94 (-11.33, -0.55) | -8.71 (-13.34, -4.08) |
| **NPC break (cm)** |  |  |
| Baseline | 6.60 (4.66, 8.53) | 6.00 (4.49, 7.52) |
| 12 weeks | 4.63 (3.36, 5.90) | 3.82 (3.10, 4.55) |
| Total change | -1.97 (-3.02, -0.92) | -2.18 (-3.48, -0.87) |
| **PFV blur or break (△)** |  |  |
| Baseline | 22.63 (19.35, 25.90) | 23.06 (20.40, 25.72) |
| 12 weeks | 34.47 (31.70, 37.24） | 35.47 (33.09, 37.85) |
| Total change | 11.84 (8.32, 15.37) | 12.41 (9.44, 15.38) |
| **Near phoria (△)** |  |  |
| Baseline | -16.31 (-18.44, -14.18) | -15.35 (-16.81, -13.90) |
| 12 weeks | -13.12 (-15.87, -10.38) | -9.94 (-13.20, -6.69) |
| Total change | 3.19 (0.88, 5.50) | 5.41 (2.40, 8.42) |

CISS: Convergence Insufficiency Symptoms Survey, NPC: near point of convergence, PFV: positive fusional vergence, △: prism diopters.

**Table 2. Outcome measures of patients with accommodative dysfunction at 12 weeks by treatment group**

| **Outcome measure** | **VR group (95% CI)** | **OBVAT group (95% CI)** |
| --- | --- | --- |
| **Monocular accommodative amplitude, (D)** |  |  |
| Baseline | 10.94 (7.80, 14.08) | 10.41 (8.19, 12.64) |
| 12 weeks | 13.86 (11.05, 16.66) | 15.15 (12.82, 17.47) |
| Total change | 2.91 (0.87, 4.96) | 4.73 (1.55, 7.91) |
| **Monocular accommodative facility, (cpm)** |  |  |
| Baseline | 6.31 (4.17, 8.46) | 4.94 (1.22, 8.65) |
| 12 weeks | 16.81(10.67, 22.95) | 17.25 (15.10,19.41) |
| Total change | 10.50 (5.49, 15.51) | 12.31 (9.34, 15.28) |

D: diopters; cpm: cycles per minute
